# Can screening for low vitamin D levels prevent bone health complications in paediatric oncology patients?

**DOI:** 10.1002/cnr2.1534

**Published:** 2021-10-26

**Authors:** Leonie Naeije, Mandy Pohlui de Silva, Paul Hofman

**Affiliations:** ^1^ Department of Paediatric Oncology and Haematology Starship Children's Health Auckland New Zealand; ^2^ Liggins Institute University of Auckland Auckland New Zealand

**Keywords:** long‐term follow‐up, osteopenia, paediatric oncology, supportive care, vitamin D

## Abstract

**Background:**

No international standards include vitamin D levels at diagnosis or during treatment. It is included in the Children's Oncology Group long‐term follow‐up guidelines. However, bone health complications (like osteopenia and atraumatic fractures) can occur at diagnosis or during treatment as well.

**Cases:**

In this small case series, we illustrate the complexity of bone health complications among our broad paediatric oncology population. If the vitamin D level is low we supplement the patient with one standard oral dose (150 000 units for 1–2 year olds, 300 000 units for 2–5 year olds and 600 000 units for >5 year olds). We do not adjust depending on diagnosis.

**Conclusion:**

Because of the potentially negative outcomes on short, medium and long term, we recommend checking vitamin D levels on diagnosis for all newly diagnosed patients. It is a simple, low cost test and one dose of oral supplementation can easily treat the deficiency.

## 1 INTRODUCTION

Children receiving cancer treatment are at high risk for reduced bone mineralization (or osteopenia), resulting in osteoporosis and secondary complications such as vertebral fractures, or even osteonecrosis. The aetiology of the reduced bone mineralization is multifactorial. It includes a direct effect of the disease itself, treatment effects (especially high dose glucocorticoid therapy), reduced sun exposure, decreased nutrition and immobility and hormonal therapy to suppress menstruation.^1^, ^2^

Bone formation is affected by gender, the hormonal environment, nutritional status, vitamin D, weight‐bearing exercise and genetics. Strong links exist between reduced bone mineral density (BMD) and vitamin D levels.^3^, ^4^

Vitamin D is converted to 1:25 cholecalciferol where it acts via the vitamin D receptor. The increased absorption of calcium and phosphate from the gut is the most important action for improving bone health and reducing osteopenia. All cancer therapies can decrease BMD through long‐term endocrine alterations and altered body composition. Endocrine, metabolic, and skeletal sequelae are among the most frequently described complications in long‐term survivors, affecting between 20% and 50% of the patients.^5^ Testing a vitamin D level is simple and supplementation can be given in one oral dose.^6^ We will illustrate the complexity and heterogeneity of bone health complications and the relation with vitamin D levels and review the literature.

## 2 CASES

### 2.1 Case 1

A 3.5‐year‐old Samoan girl diagnosed with B cell acute lymphatic leukaemia (ALL), started treatment per COG AALL0932 was found to have an insufficient 25‐OH vitamin D level 3 weeks after she was diagnosed. The 25‐OH vitamin D level was 37 nmol/L (normal range 50–150 nmol/L). Due to febrile neutropenia and a clinical suspicion of osteomyelitis, an X‐ray of tibia and fibula were made showing diffuse osteopenia. She received supplementation of vitamin D per local protocol, 300 000 IU in one oral dose. Despite this supplementation 4 months later the vitamin 25‐OH vitamin D level was 19 nmol/L, and supplementation of 300 000 IU was given per local protocol. The level was checked 2 months later and was found to be sufficient. The patient had entered the maintenance phase of her ALL treatment at that stage.

### 2.2 Case 2

A 4‐year‐old New Zealand European girl, diagnosed with T cell ALL, received treatment per COG AALL0434, high‐risk arm, including cranial radiation at 12 Gy. The treatment was complicated by a PICU admission due to sepsis with cardiac arrest, renal failure and typhlitis with ileus and intravenous nutrition dependence due to a pseudomonas bacteraemia.

During maintenance therapy she presented with back and leg pain and was found to have an insufficient 25‐OH vitamin D level (30 nmol/L). A DEXA scan revealed a *Z*‐score of −3.6 and more imaging revealed extensive central vertebral body collapse in her spine, particularly in the lower thoracic and lumbar spine, as well as osteopenic bones on X‐ray. She was treated for her osteopenia as per the local endocrine team with supplementation of vitamin D (300 000 IU oral dose once) and six monthly Zoledronate, 1 mg/kg/dose. The DEXA scan repeated 5 year later showed a *Z*‐score of +1.3 (normal) and she was clinically asymptomatic.

### 2.3 Case 3

A 8‐year‐old New Zealand European boy, diagnosed with medulloblastoma, received chemotherapy and radiotherapy per SJMB03 protocol. His treatment was complicated by severe acquired brain injury, resulting in immobility. At the end of his treatment, he had a fall from his bed and investigations revealed osteopenia and a transverse fracture of the distal femur. The 25‐OH vitamin D level was 52 nmol/L. He was started on calcium supplements per the local endocrine team. Follow‐up vitamin D level was 75 nmol/L 5 months later.

### 2.4 Case 4

A 17‐year‐old New Zealand European boy, was diagnosed with stage 2a Hodgkin Lymphoma, treated per EURONET protocol, no radiation. He was diagnosed with avascular necrosis at the end of his treatment on MRI, 25‐OH vitamin D level was 50 nmol/L. He was also diagnosed with muscular weakness due to steroids; this improved over follow‐up.

### 2.5 Case 5

A 16‐year‐old Samoan boy, treated for BCOR Sarcoma per AEWS0031 protocol. His treatment was complicated by multiple fractures due to trauma and osteopenia. His 25‐OH vitamin D level was <10 nmol/L and supplemented according to the local protocol with one single oral dose of 600 000 IU. Hereafter, he was lost to follow‐up.

## 3 DISCUSSION

The heterogeneity and complexity of low levels of vitamin D and bone health complications is illustrated here. Adequate vitamin D levels alone will not prevent all the bone health complications in this patient population; these complications are multifactorial. However, the size of adequate vitamin D levels during treatment or at the diagnosis has not been investigated and no published international guidelines or recommendations were found. Limited research is done to investigate the importance of bone health complications in this population during treatment, most published studies report about childhood cancer survivorship patients.^5^ Prevalence of vitamin D deficiency in paediatric oncology patients has been reported between 37%^7^ and 70%.^8^ Vitamin D deficiency is known to be more prevalent in people with a darker skin or when the skin has limited sun exposure, malnutrition can also play a role.

Orgel et al. focussed specifically on bone changes during induction treatment in patients with ALL. No significant differences were found in assessment pre‐treatment compared to healthy individuals. After the induction phase, containing steroids, a significant decline was seen in the cancellous vertebral BMD, representing a median loss of 26.8%. The majority of the patients had insufficient or deficient levels of vitamin D (<20 ng/mL) at diagnosis (66%).^9^

The STeroid‐association Osteoporosis in the Pediatric Population (STOPP) research initiative is an extensive Canadian paediatric bone health research program.^2^, ^10^, ^11^ Newly diagnosed paediatric patients with ALL were enrolled and had a baseline bone assessment. Lumbar spine BMD (LS BMD) *Z*‐score was reduced for the entire cohort when determined based on chronological age and bone age.^10^ In 20% of the patients a vertebral fracture was diagnosed in the first 4 years after the diagnosis, the majority of those patients were asymptomatic.^11^

Currently, it is not standard to include vitamin D levels or BMD studies at diagnosis or during treatment for children diagnosed with a malignancy to assess their bone health. However, it is included in the Children's Oncology Group (COG) long‐term follow‐up guidelines. They recommend an initial evaluation of BMD at entry into long‐term follow‐up; acknowledging hereby that childhood cancer survivors are at risk for these bone health complications.^12^

In this brief report, we present a small heterogenetic case series. It illustrates the range of possible bone health complications in the broad spectrum of paediatric oncology during treatment. We recognize that not all bone health complications are solely related to low vitamin D levels, and have a multifactorial aetiology. However, we do feel that an adequate vitamin D level maintained during treatment can prevent complications.

We acknowledge that an adequate vitamin D level will not result in the complete normalization of bone mineralization in our patient population.

Long‐term outcome data are not yet available to support the benefit of an optimized vitamin D level during treatment. However, vitamin D deficiency is common and may result in negative bone health outcomes in the short, medium and long term for paediatric oncology patients. Given the potentially negative outcomes, the ease of treatment and its low cost, vitamin D deficiency should be identified and treated aggressively on presentation. We recommend prospective long and medium term studies to investigate the benefits for adequate vitamin D levels in paediatric oncology patients.

## CONFLICT OF INTEREST

All authors have no conflict of interest.

## AUTHOR CONTRIBUTIONS

All authors had full access to the data in the study and take responsibility for the integrity of the data and the accuracy of the data analysis. *Conceptualization*, L.N., M.P.D.S., P.H.; *Writing—Original Draft*, L.N.; *Writing—Review & Editing*, L.N., M.P.D.S., P.H.; *Supervision*, P.H.; *Data Curation*, L.N.

## ETHICAL STATEMENT

We received institutional approval for the retrospective study with no need for patients or parents consent.

## Data Availability

Data sharing is not applicable to this article as no new data were created or analyzed in this study. n/a
